# A high-efficiency automatic pressure-relief drug transfer device for anticancer medications with superior closed performance

**DOI:** 10.3389/fphar.2025.1579771

**Published:** 2025-04-25

**Authors:** Liming Xie, Guanfeng Chen, Qianqian Ouyang, Weiyan Quan, Xiaoming Xie, Xiaoling Chen, Lefan Li, Sidong Li, Rizhi Chen, Rongqiong Luo, Zhiqing Qiu

**Affiliations:** ^1^ Department of Pharmacy, The Affiliated Hospital of Guangdong Medical University, Zhanjiang, China; ^2^ School of Ocean and Tropical Medicine, Guangdong Medical University, Zhanjiang, Guangdong, China; ^3^ School of Chemistry and Environmental Science, Guangdong Ocean University, Zhanjiang, China; ^4^ Guangdong Jianliyuan Medical Technology Co., Ltd., Zhanjiang, China

**Keywords:** closed performance, automatic pressure-relief, hazardous drugs, closed-system drugtransfer device, occupational protection, CSTD

## Abstract

**Objective:**

Preventing exposure to hazardous drugs is crucial for healthcare workers to avoid health risks. To mitigate healthcare workers’ risks when handling hazardous drugs, we developed a novel closed-system drug-transfer device (CSTD) called CSTD(JLY). This CSTD has an automatic pressure-relief structure. Hence, it can significantly decrease the resistance in the push/pull of a piston rod if an operator transfers drugs, thereby reducing the burden on the hands of the operator during drug transfer.

**Methods:**

We investigated the closed performance of the novel CSTD (JLY) by comparing it with the performance of a syringe. We selected a simulation drug (fluorescein sodium), commonly used drugs (lansoprazole, nimodipine, and tropisetron), and a commonly used anti-cancer agent (cyclophosphamide) to conduct exposure evaluation.

**Results:**

Compared with a syringe, CSTD(JLY) could reduce drug leakage significantly. Our novel CSTD with an automatic pressure-relief structure had superior closed performance. CSTD(JLY) could solve the problem of liquid-drug leakage during drug transfer.

**Conclusion:**

This feature could reduce the exposure risk healthcare workers and patients.

## 1 Introduction

Concentrated drug transfer at pharmacy intravenous admixture services (PIVAS) is an important measure for hospitals to improve the safety, efficiency, and normalization of drug treatment. However, the safety at PIVAS faces multiple challenges, especially for the transfer of anti-cancer drugs (ACDs).

Most ACDs are cytotoxic preparations with teratogenic, carcinogenic, and mutagenic effects. They cause damage to normal human tissues and organs, especially to bone marrow, the digestive tract and reproductive system, while killing or inhibiting tumor cells (Moretti et al., 2011; Vainio, 1982). Moreover, nurses or pharmacists participating in the compounding, storage, or transport of ACDs are exposed to the leakage of toxic drugs (Brechtelsbauer, 2023; Zhu et al., 2023). Numerous studies have demonstrated that long-term and frequent exposure to ACDs may have far-reaching effects owing to accumulation within the body. Such accumulation can result in occupational hazards to healthcare workers: bone-marrow suppression, leukopenia, hair loss, gastrointestinal symptoms, skin allergies, corneal damage, damage to chromosomes and DNA, and menstrual abnormalities (Sorsa et al., 1985; Sorsa and Anderson, 1996).

The operation used most frequently at a PIVAS is drug transfer by a syringe (Sessink et al., 2011), which has the advantage of low cost, but the operator must exert significant force to transfer the liquid drug from a vial. Long-term operation can cause tenosynovitis in the hand (Nimunkar et al., 2017). More importantly, syringe use has a poor closed performance. In particular, a liquid drug can overflow during drug transfer, so drug leakage is possible (Fox et al., 2023; Yang et al., 2023; Hao et al., 2022). Studies have demonstrated a prevalence of contamination of 0.42% in the suction of liquid drugs through regular operation by a syringe (Miyake et al., 2013). A PIVAS can achieve the transfer of toxic drugs for chemotherapy in a zonal, concentrated manner in a negative-pressure environment using a “biological safety cabinet”. This strategy greatly alleviates the risk of healthcare workers being exposed to ACDs. However, drug transfer using a syringe at a PIVAS increases the probability of ACD exposure (Alavattam et al., 2020; Marler-Hausen et al., 2020; Amichay et al., 2021).

In 2004, the National Institute for Occupational Safety and Health (NIOSH) detailed a drug-transfer device that mechanically prohibits the transfer of environmental contaminants into the system and the escape of hazardous drug or vapor concentrations outside a closed-system drug-transfer device (CSTD) (Bartel et al., 2018). Compared with use of a syringe in conventional transfer of a drug, a CSTD can prohibit external environmental agents from contaminating drugs or drugs leaking into the environment (Harrison et al., 2006). This device makes the entire process of drug transfer complete in a relatively closed condition. This device reduces direct exposure of healthcare workers to chemotherapy drugs, as well as improving the safety and efficiency of drug transfer. With a CSTD, drug transfer can be undertaken without a biological safety cabinet, so the convenience of drug transfer is improved. Studies (Harrison et al., 2006) have shown that, in an appropriate environment, a CSTD can stop drugs being contaminated within 168 h.

Multiple institutions, including the NIOSH, International Society of Oncology Pharmacy Practitioners, American Society of Health-System Pharmacists, United States Pharmacopoeia, and Oncology Nursing Society, have recommended use of a CSTD in the management of hazardous drugs. Some countries and regions have mandated the use of a CSTD by medical institutions through legislation (Ezquer-Garin et al., 2019; Chan and Lim, 2016).

Dr. Sessink developed a CSTD in 1999 called PhaSeal^®^, which was trialed for 1 year in Ängelholm Hospital in Sweden. PhaSeal prevented environmental contamination during drug transfer (Sessink et al., 1999). PhaSeal was popularized in 30 hospitals in the USA from 2004 to 2010, and comparative analysis was conducted for surface contamination of the ACD cyclophosphamide. Compared with the standard method of drug transfer, the method using the CSTD PhaSeal caused the surface contamination of cyclophosphamide to decrease from 95% to 13% (Sessink et al., 2013). Favier et al. (2012) compared the environmental contamination before and after the introduction of PhaSeal into the pharmacies of two hospitals. When PhaSeal was used for drug transfer, contamination of the work environment decreased by 93%. Clark and Sessink (2013) conducted sampling by zone at 12 locations, and studied the contamination by cyclophosphamide and 5-fluorouracil in a hospital after use of the CSTD EquaShield^®^. Contamination by cyclophosphamide and 5-fluorouracil was not found in any of the pharmacies, infusion rooms, or cancer center office. Bartel et al. (2018) reported on the effectiveness of the CSTD Halo^®^ used in ACD transfer and administration processes on the reduction in surface contamination by sampling at 13 locations in oncology hospitals in the USA. At least one marked ACD was detected in 66.7% of samples (104 out of 156 samples) before use of the CSTD. The percentage of samples contaminated decreased to 5.8% after use of the CSTD (9 out of 156 samples). In the administrative zones, the percent contamination was 78% (61 out of 78 samples) before use of the CSTD, and only 2.6% after use of the CSTD. In addition, 26 participants provided scores for the ease of use of the CSTD: all of them expressed that they were “quite satisfied” or “very satisfied” with the CSTD. Yu et al. (Gerding et al., 2022) used PhaSeal in China to evaluate the effect of a CSTD used at a PIVAS on the cyclophosphamide contaminant residue in a work environment. They took samples from 19 positions at worktops, transfer trolleys, and platforms for leaving cabins in three biological safety cabinets. When the conventional method of drug transfer was used, the median value of all monitoring points was 1.30 ng/cm^2^ before cleaning and decontamination, and 0.22 ng/cm^2^ after cleaning and decontamination. After the CSTD had been used for drug transfer, the median value of all monitoring points was 0.06 ng/cm^2^. Therefore, use of a CSTD can reduce the leakage of hazardous drugs during compounding and transfer.

The global manufacturers producing CSTDs are mainly BD Medical, Equashield, and ICU Medical. These three manufacturers collectively hold >80% of the market share. North America accounts for ∼75% of the market share, followed by Europe and Japan (∼20%) (Alavattam et al., 2020; Tang et al., 2022; Hilliquin et al., 2019).

CSTDs have not been popularized widely in the Chinese market, but some Chinese enterprises have seen business opportunities and developed them. From the perspective of product design, if a CSTD for the transfer of ACDs is developed and launched, it will have a broad market space (Hao et al., 2022; Piccardo et al., 2021; Wilkinson et al., 2024). However, owing to high cost of the CSTD (such as BD Medical, Equashield, and ICU Medical, etc.), it cannot be fully popularized in most regions in the world.

We wished to reduce potential occupational hazards to healthcare workers in the transfer of ACDs. Our research team developed a novel, dual-barreled CSTD called “CSTD(JLY)” in 2015. We obtained Chinese invention patent authorizations for relevant technologies in 2020 (Xie et al., 2021; Chen et al., 2020; Chen et al., 2023a; Chen et al., 2023b) and applied for European invention patents (Chen, 2024).

To investigate the performance of this novel CSTD (JLY), we chose several commonly used drugs to conduct exposure evaluation of CSTD(JLY). Our study could solve the problem of leakage upon the transfer of liquid drugs, thereby reducing the exposure risk for healthcare workers and patients.

## 2 Experimental

### 2.1 Materials

Fluorescein sodium, lansoprazole, nimodipine, tropisetron, cyclophosphamide, and physiologic (0.9%) saline, which were all analytical reagents, were purchased from Shanghai Aladdin Biochemical Technology (Shanghai, China).

**FIGURE 1:**
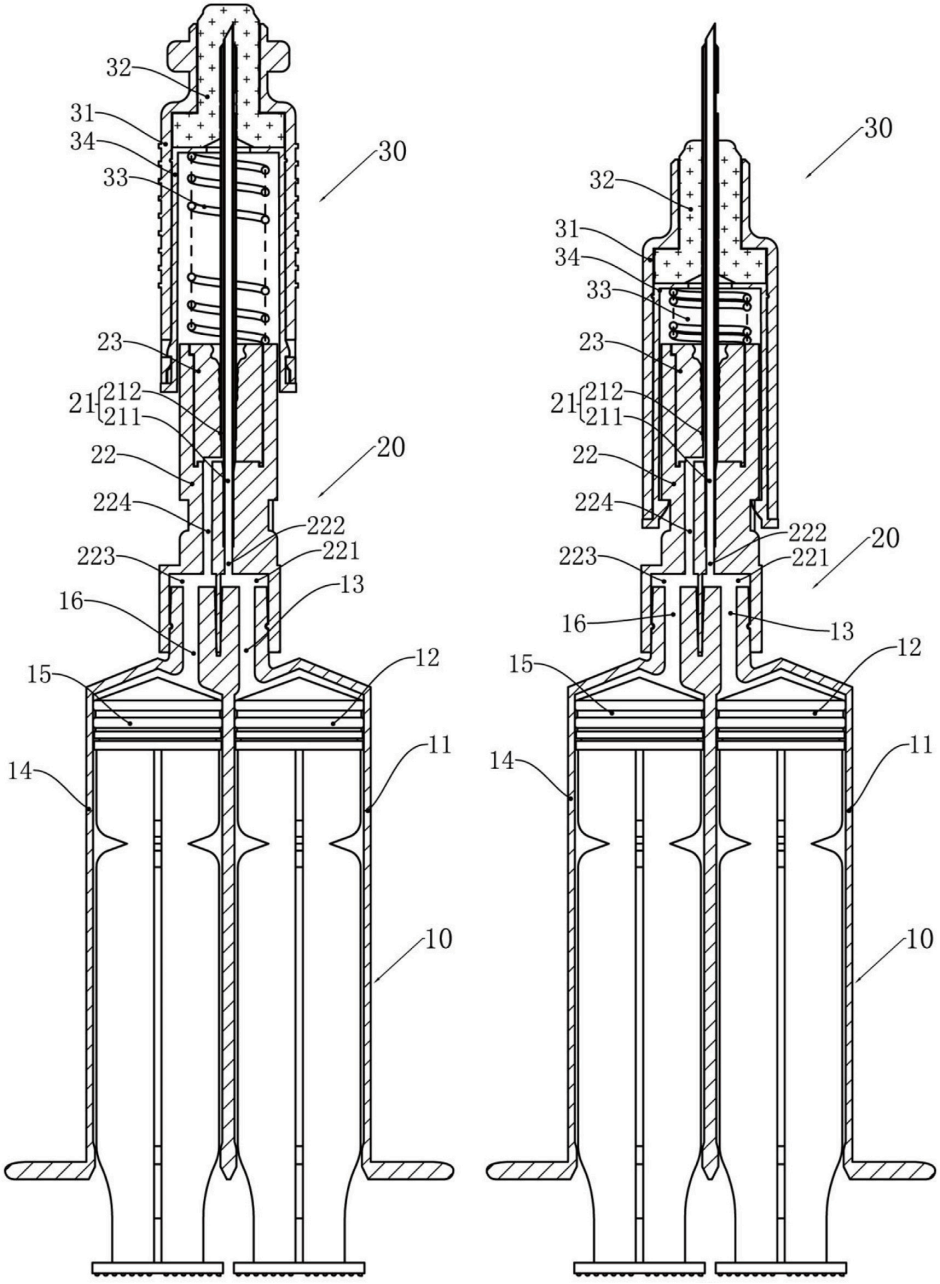
A dual-barrel CSTD called “CSTD(JLY)” (schematic). 10 = double-chamber syringe. 11 = first syringe barrel. 12 = first piston rod. 13 = first joint. 14 = second syringe barrel. 15 = second piston rod. 16 = second joint. 20 = transfer needle assembly. 21 = transfer needles. 211 = inner columnar channel. 212 = side annular channel. 22 = rear end of needle seat. 221 = first connection hole. 222 = first channel. 223 = second connection hole. 224 = second channel. 23 = needle penetrating. 30 = syringe needle sealing assembly. 31 = sheath. 32 = closing member. 33 = elastic member. 34 = inner sleeve.

The CSTD used in experiments, CSTD(JLY), was provided by Guangdong Jianliyuan Medical Technology (Guangdong, China), and its main structure is shown in Figure 1. Figure 1A shows CSTD(JLY) with a needle in a sheath. Figure 1B shows CSTD(JLY) with the needle sheath rotated by 90°. The components of CSTD(JLY) are shown in Figure 1.

### 2.2 Methods

All experiments were conducted at 25°C and 1 atmosphere of pressure. Weigh 1.00 g of the drug and put it into the vial, inject 10 mL of normal saline into the vial and dissolve the drug evenly, then seal it with a sealing cap. The concentration of all drugs was 0.100 g/mL and the volume was 10 mL.

In Experiment I, drug transfer was simulated in a laboratory. The gliding performance of the piston rod in the drug-transfer device during push/pull for drug transfer was determined with a tensile testing machine (CH-983C; Shengwu New Materials (Shenzhen), Shenzhen, China). A syringe and CSTD(JLY) were compared for differences. All data were presented in this study as the mean ± standard deviation (SD) of three independent experiments.

In Experiment II, drug transfer was simulated in a laboratory. A syringe and CSTD(JLY) were compared for differences in percent drug leakage during the transfer of fluorescein sodium (FS). The drugs in the infusion bottle, drug-transfer device, puncture outfit, and vial were collected separately. Collect the liquid directly from the infusion bottle, and then add 10 mL of solvent to flush the infusion bottle after collection, and finally collect it into the liquid medicine. This part serves as the final drug utilization part. Rinse the dispenser and the cillin bottle with 10 mL of solvent, respectively, and collect the residue as the dispenser and the residue of the Cillin bottle.FS content (mg/mL) in all of the parts exposed to the drug were determined with a high-performance liquid chromatography (HPLC) instrument (LC-40; Shimadzu, Beijing, China) to indirectly investigate FS leakage. The quality of the drug was calculated. Finally, mass loss is used to represent. In addition, FS leakage was directly shown by photography under irradiation by an ultraviolet lamp.

In Experiment III, the transfer of three drugs (lansoprazole, nimodipine, tropisetron) was simulated in a laboratory. A syringe and CSTD(JLY) were compared for differences in drug leakage during drug transfer. The drugs in the infusion bottle, drug-transfer device, puncture outfit, and vial were collected separately. Collect the liquid directly from the infusion bottle, and then add 10 mL of solvent to flush the infusion bottle after collection, and finally collect it into the liquid medicine. This part serves as the final drug utilization part. Rinse the dispenser and the cillin bottle with 10 mL of solvent, respectively, and collect the residue as the dispenser and the residue of the Cillin bottle. The drug content in all of the parts exposed to the drug were determined with a HPLC instrument (LC-40) to investigate drug leakage. The quality of the drug was calculated. Finally, mass loss is used to represent.

In Experiment IV, the ACD cyclophosphamide was transferred in a biological safety cabinet at the PIVAS of the Affiliated Hospital of Guangdong Medical University (Guangdong, China). A syringe and CSTD(JLY) were compared for differences in drug leakage during drug transfer. The drugs in the infusion bottle, drug-transfer device, puncture outfit, and vial were collected separately. Collect the liquid directly from the infusion bottle, and then add 10 mL of solvent to flush the infusion bottle after collection, and finally collect it into the liquid medicine. This part serves as the final drug utilization part. Rinse the dispenser and the cillin bottle with 10 mL of solvent, respectively, and collect the residue as the dispenser and the residue of the Cillin bottle. The drug content in all of parts exposed to the drug were determined with a HPLC instrument (LC-40) to investigate drug leakage. The quality of the drug was calculated. Finally, mass loss is used to represent.

In Experiments II–IV, all data were presented in this study as the mean ± standard deviation (SD) of six independent experiments, percent leakage was calculated using the following formula:

$$\text{Yield in infusion bottle}=\frac{m1}{m0}\times 100\%$$

$$\text{Residual rate in drug}-\text{transfer device and puncture outfit}=\frac{m2}{m0}\times 100\%$$

$$\text{Residual rate in vial}=\frac{m3}{m0}\times 100\%$$

Leakage = 100% − Yield in infusion bottle − Residual rate in drug-transfer device and puncture outfit − Residual rate in vial.

Which m0 is the original quality of the drug before dispensing, m1 is the mass of the drug in the infusion bottle, m2 is the mass of the drug in drug-transfer device and puncture outfit, m3 is the mass of the drug in vial.

### 2.3 Statistical analysis

All data were presented in this study as the mean ± standard deviation (SD) of three to six independent experiments. Analysis of variance (ANOVA) was performed, and Tukey’s test was used to determine the significance of the differences between data with p < 0.05 as statistically significant.

## 3 Results and discussion

### 3.1 Structural characteristics of CSTD(JLY)

**FIGURE 2:**
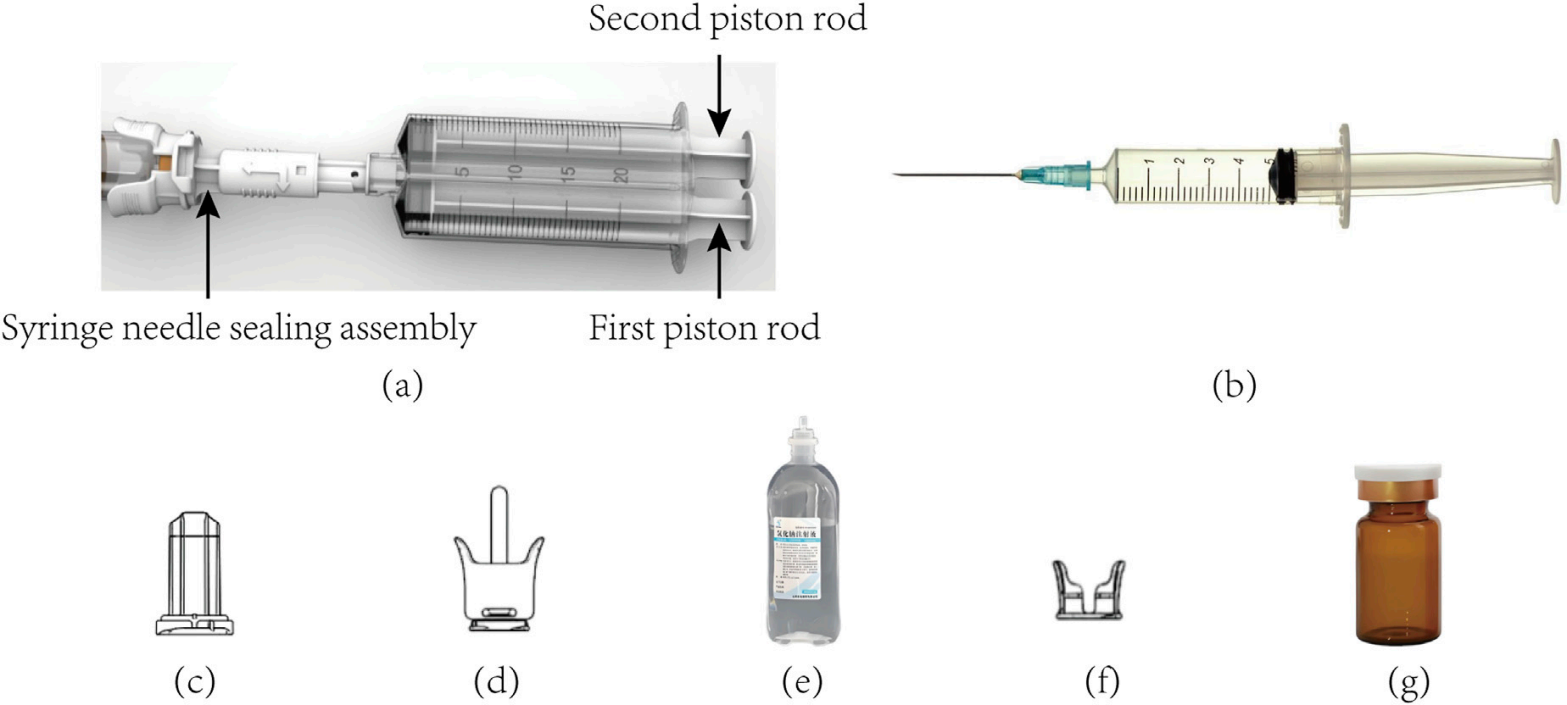
Photographs of the various parts involved in drug transfer by CSTD(JLY). **(a)** CSTD(JLY) **(b)** PS **(c)** air filter **(d)** luer/spike connector **(e)** infusion bottle **(f)** vial connector **(g)** vial.

Figure 2 shows the various parts used in drug transfer by CSTD(JLY), including the dual-barrel drug-transfer device, air filter, Luer/Spike connector, and vial connector. They were designed for ACDs to ensure no leakage during drug transfer (Chen et al., 2020; Chen et al., 2023a; Chen et al., 2023b; Chen, 2024). A detailed description based on drug transfer by CSTD(JLY) is shown below.

#### 3.1.1 System preparation and air storage

First, all parts of CSTD(JLY) were assembled correctly and in good operating condition (Figures 1, 2). The air filter was connected with the syringe needle sealing assembly (SNSA). The first piston rod was pulled outwards to store air in the first syringe barrel, making preparations for subsequent air replenishment and pressure relief. After air storage had been completed, CSTD(JLY) was disconnected from the air filter.

#### 3.1.2 Suction of solvent

First, The Luer/Spike connector was inserted into the infusion bottle. The other end was connected firmly with the SNSA.

Second, the needle sheath was rotated by 90° (Figure 1). This action caused the needle to pierce the sealing assembly and stretch into the infusion bottle. At this moment, the needle sealing assembly came into close contact with the infusion bottle, maintaining a closed state during liquid suction.

Third, the second piston rod was pulled to suck one part of the solvent (e.g., 0.9% saline) into the second syringe barrel from the infusion bottle through a closed channel. At this moment, negative pressure was generated in the infusion bottle, which can result in leakage. Thus, the air stored in the first syringe barrel was replenished automatically to the infusion bottle until a balance in air pressure was reached, ensuring that the liquid was strictly closed within this system during liquid suction.

#### 3.1.3 Dissolution of drug powder and suction of a liquid drug

First, after solvent suction had been completed, the SNSA was rotated by 90° so that the needle retracted into the SNSA (Figure 1). Then, CSTD(JLY) was disconnected with the Luer/Spike connector. At this moment, the infusion bottle maintained a closed connection with the Luer/Spike connector.

Second, one end of the vial connector was connected with the vial and the other end was connected with the SNSA. At this moment, the front-end face (with sealing rubber) of the SNSA was closely connected with the rubber opening of the vial, so that the whole system was closed. CSTD(JLY) was rotated by 90°, causing the needle to pierce the sealing assembly and stretch into the vial. Then, the second piston rod was pushed to inject the solvent into the vial, which was used to dissolve the drug powder.

Third, after the drug powder in the vial had dissolved fully, the liquid drug was sucked into the second syringe barrel. At this moment, negative pressure was generated in the vial, which can result in leakage. Thus, the air stored in the first syringe barrel was replenished automatically to the vial, ensuring that the liquid was strictly closed within this system in the entire process.

#### 3.1.4 Transfer of liquid drug

First, after suction of the liquid drug had been completed, the SNSA was rotated by 90° so that the needle retracted into the SNSA. Then, the drug-transfer device was rotated reversely so that CSTD(JLY) was disconnected with the vial connector.

Second, the SNSA was connected with the Luer/Spike connector on the infusion bottle. At this moment, the front-end face (with sealing rubber) of the SNSA was closely connected to the rubber opening of the Luer/Spike connector. The dual-barrel drug-transfer device was rotated, causing the needle to pierce the sealing assembly and stretch into the infusion bottle. Then, the second piston rod was pushed to transfer the liquid drug to the infusion bottle. At this moment, positive pressure was generated in the infusion bottle, which can result in leakage. However, air pressure can cause automatic discharge of air into the first syringe barrel, ensuring that the liquid was strictly closed within this system in the whole process.

#### 3.1.5 Drug transfer by a syringe

At present, drug transfer by syringe is used mainly clinically. A syringe is inserted into the infusion bottle and sucks the solvent, and then the solvent is injected into the vial. After the drug powder is dissolved, the liquid drug in the vial is sucked and injected into the infusion bottle. There is no strict sealing or timely positive/negative pressure relief in each operation, so leakage of a liquid drug is serious.

Compared with a syringe, if CSTD(JLY) is used for drug transfer, the transfer needle is reset automatically and resealed after each operation in the entire process. The connection in each step of drug transfer reaches a strict closed state, and positive/negative pressure in the vial and infusion bottle is relieved in time. This process ensures that exposure to and contamination of a liquid drug does not occur. Hence, the safety of ACD transfer is greatly increased, which alleviates the risk to healthcare workers.

### 3.2 Gliding performance of CSTD(JLY) and a syringe

The gliding performance of a drug-transfer device directly affects the safety and effectiveness of drug transfer. A drug-transfer device with good performance should ensure neither injection difficulty owing to too great a resistance nor incorrect delivery of a drug owing to too low a resistance during push/pull. Good gliding performance can ensure smoothness during use, thus reducing the leakage caused during drug transfer and enhancing user comfort.

**FIGURE 3:**
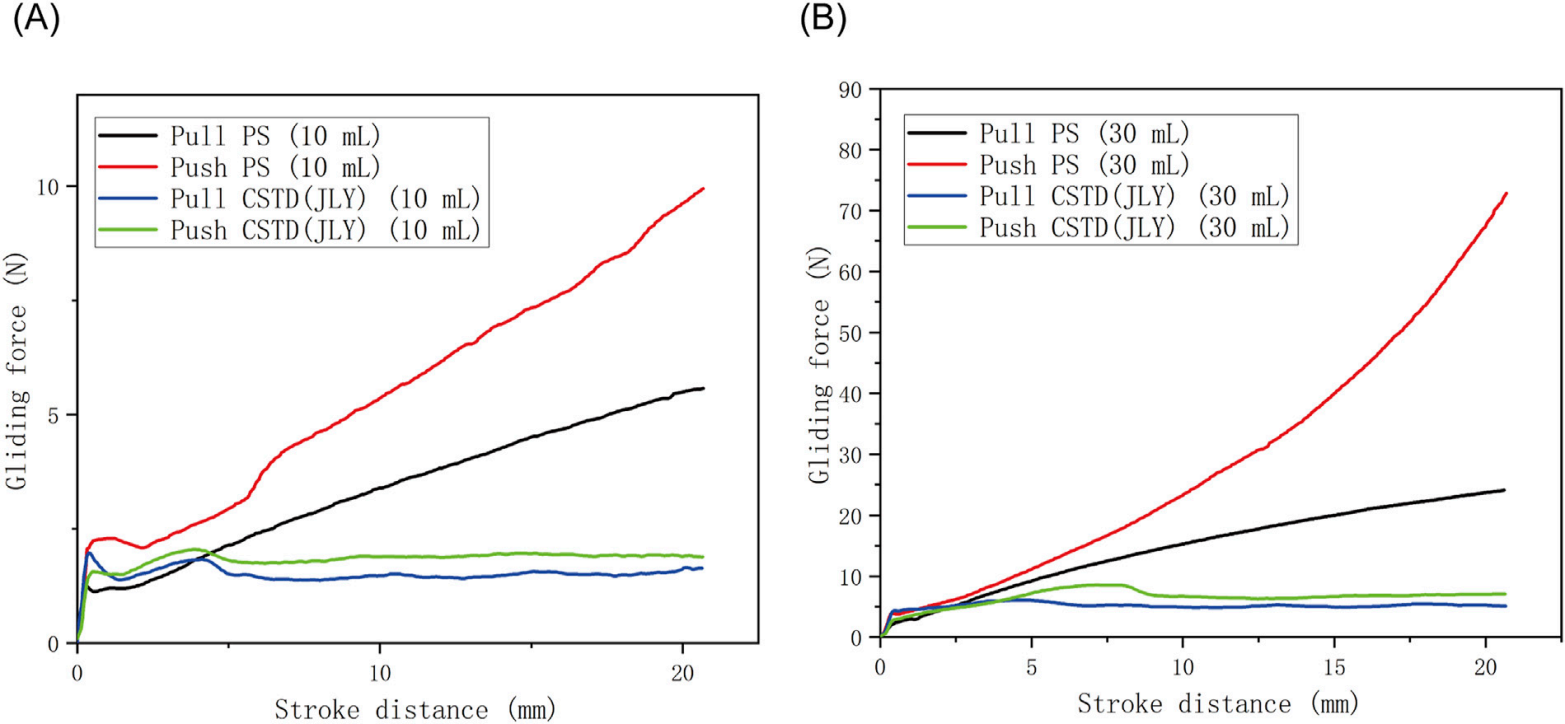
Comparison of push/pull gliding performance between a syringe (PS) and CSTD(JLY) of sizes **(A)** 10 mL and **(B)** 30 mL.

In this experiment, two sizes (10 mL, 30 mL) of CSTD(JLY) were investigated for gliding performances during pull and push, with comparison with a syringe being conducted (Figure 3; Table 1).

**TABLE 1:** Maximum push/pull gliding forces of PS a syringe and CSTD(JLY) of different sizes (n = 3).

Drug-transfer device	Gliding force (N)	
CSTD(JLY)	10 mL (pull)	1.7 ± 0.16
	10 mL (push)	2.4 ± 0.23
	30 mL (pull)	6.3 ± 0.29
	30 mL (push)	8.6 ± 0.76
PS	10 mL (pull)	5.5 ± 0.52
	10 mL (push)	8.0 ± 0.54
	30 mL (pull)	23.5 ± 2.04
	30 mL (push)	76.35 ± 4.07

According to Figure 3, in a push/pull stroke of 0–21 mm, the maximum gliding force in a push/pull process of a 10-mL drug-transfer device was smaller than that in the push/pull process of a 30-mL drug-transfer device. Additionally, for both devices, the push gliding force tended to be larger than the pull gliding force. The push/pull gliding force of CSTD(JLY) tended to be stable after 5 mm of gliding.

The maximum gliding force in the push/pull of a syringe was far greater than that in the push/pull of CSTD(JLY) (Table 1). With an increase in the push/pull stroke, the gliding force exhibited a linear ascending trend, reaching 5.5–76.35 N at 21 mm, which was about 3–9-times the gliding force (1.7–8.6 N) of CSTD(JLY). The latter had a relatively even gliding force, maintained at ∼2 N (10 mL) and ∼6 N (30 mL). The air pressure generated in the push/pull of piston rod in one of the syringe barrels is transferred to the other syringe barrel (this is why air storage is required; see Section 3.1.1). Hence, the air pressure is balanced and the force used by the operator is small and even during the entire push/pull process.

These results suggested that CSTD(JLY) could decrease the resistance in the push/pull of the piston rod by the operator during drug transfer. This phenomenon greatly reduced the flowing pressure of the liquid drug, thus preventing the risk of liquid-drug leakage. If a syringe is used, the operator must use a large force repeatedly in the entire operation to complete drug transfer. This action causes muscle fatigue and joint pain in the hands, which can result in tenosynovitis.

### 3.3 Closed performance of CSTD(JLY)

We chose several commonly used clinical drugs to conduct exposure evaluation for CSTD(JLY) and a syringe.

#### 3.3.1 Closed performance of CSTD(JLY) for FS transfer

**FIGURE 4:**
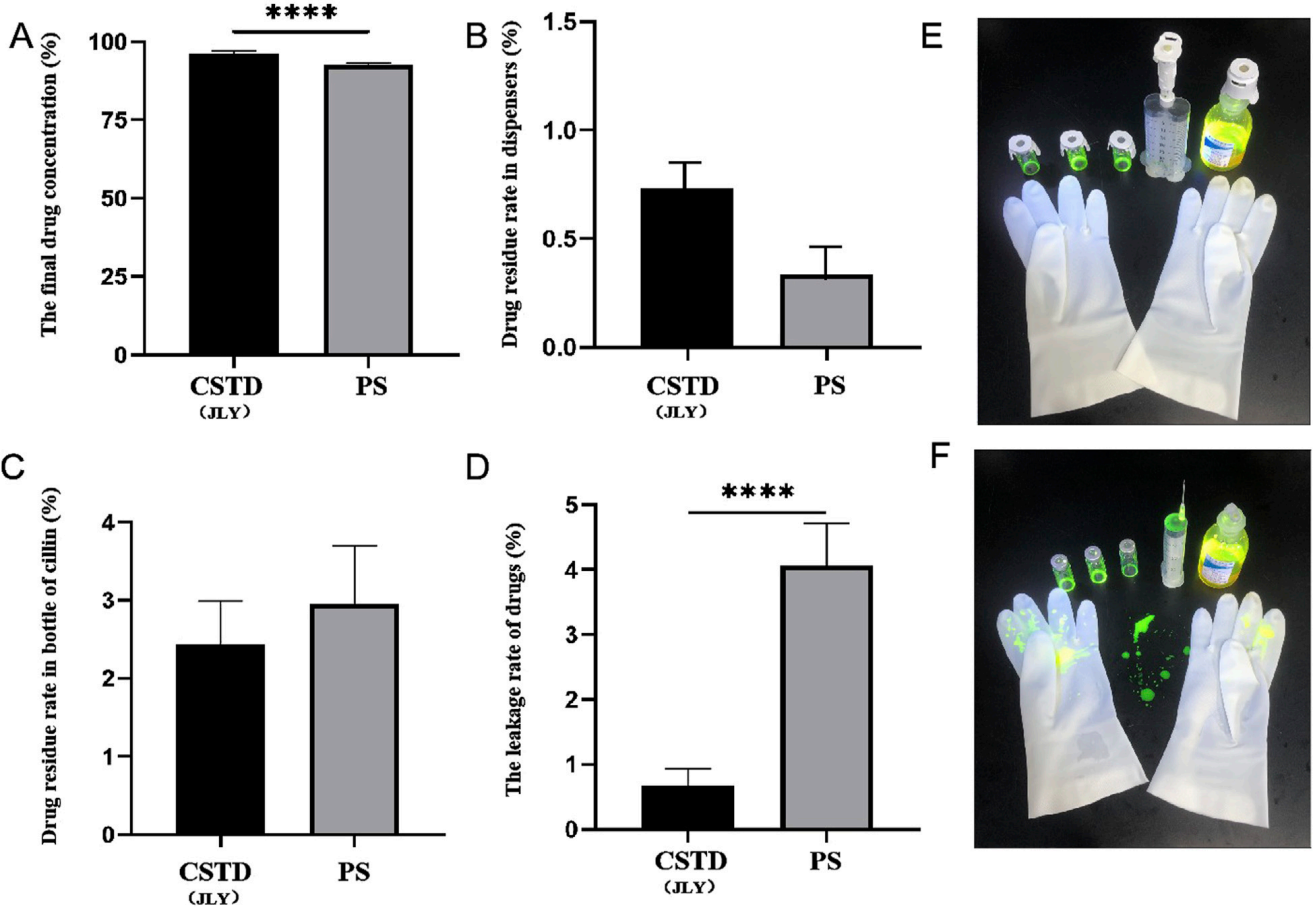
Closed performance of CSTD(JLY) and a syringe (PS) for the transfer of fluorescein sodium (FS): **(A)** Final concentration of FS in an infusion bottle; **(B)** Percent residual of FS in the drug-transfer device; **(C)** Percent residual of FS in a vial; **(D)** Percent leakage of FS; **(E)** FS leakage after transfer by CSTD(JLY); **(F)** FS leakage after transfer by a PS; Statistically significant differences from the syringe group were indicated as ****p < 0.0001.

The average yield of drug in an infusion bottle was 96.17% upon drug transfer by CSTD(JLY) and 92.64% in the drug-transfer process by a syringe (Figure 4A). Compared to the syringe, drug utilization upon drug transfer by CSTD(JLY) was significantly higher than that upon drug transfer by a syringe. During drug transfer by CSTD(JLY), the drug transfer and puncture outfit were flushed separately repeatedly to collect the drugs from them. The collected drugs were added together to obtain a residual sample for CSTD(JLY). Figure 4B reveals that the average percent residual was 0.73% in upon drug transfer by CSTD(JLY) and 0.33% upon drug transfer by a syringe, and the difference was not significant. Hence, the average percent residual was equivalent between these two drug-transfer devices. Figure 4C reveals that the percent residual in a vial was 2.44% upon drug transfer by CSTD(JLY) and 2.96% upon drug transfer by a syringe, and the difference was not significant. Hence, the average residual in a vial was equivalent between these two drug-transfer methods. As shown in Figure 4D, the average percent leakage of a sample upon drug transfer by CSTD(JLY) (0.67%) was significantly lower than that upon drug transfer by a syringe (4.06%).

Figure 4E shows photographs under irradiation by a ultraviolet lamp when the analyst wore gloves to transfer FS using CSTD(JLY). The upper part shows three vials, one CSTD(JLY), and one infusion bottle, and the lower part shows one pair of gloves. FS is yellowish-green under irradiation. Figure 4E reveals no FS leakage after drug transfer by CSTD(JLY).

Figure 4F shows photographs under irradiation by a ultraviolet lamp when the analyst wore gloves to transfer FS using by a syringe. The upper part shows three vials, one syringe, and one infusion bottle, and the lower part shows one pair of gloves. Figure 4F reveals serious leakage FS on the gloves and experimental-table surface after drug transfer by a syringe.

#### 3.3.2 Closed performance of CSTD(JLY) for the transfer of lansoprazole

**FIGURE 5:**
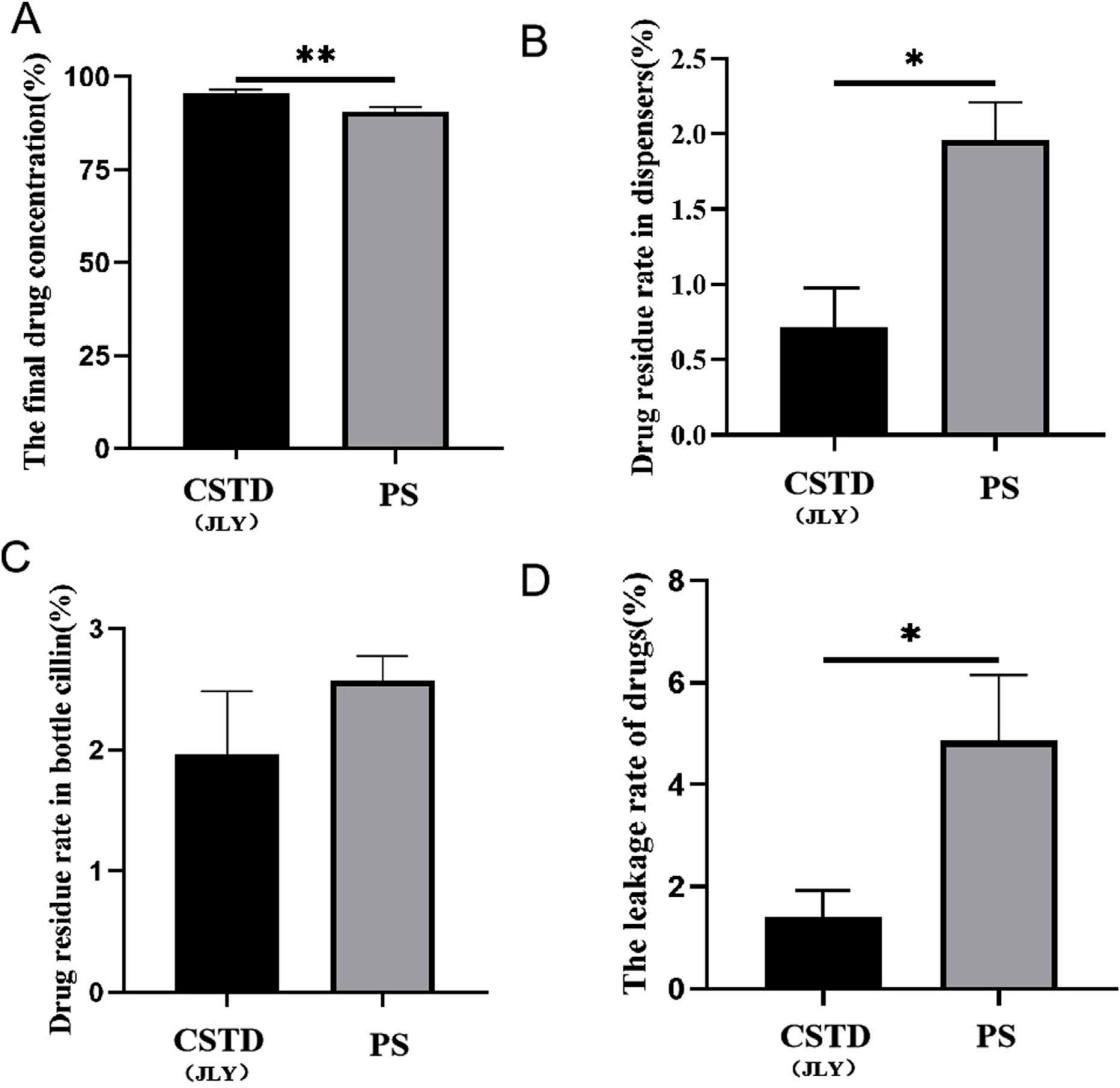
Closed performance of CSTD(JLY) and a syringe (PS) for the transfer of lansoprazole: **(A)** Final concentration of lansoprazole in an infusion bottle; **(B)** Percent residual of lansoprazole in the drug-transfer device; **(C)** Percent residual of lansoprazole in a vial; **(D)** Percent leakage of lansoprazole; Statistically significant differences from the syringe group were indicated as *p < 0.05 and **p < 0.01.

Lansoprazole was transferred by CSTD(JLY) and a syringe separately (Figure 5). The average yield of a sample in an infusion bottle upon drug transfer by CSTD(JLY) was 95.69%, significantly higher than that of a sample in an infusion bottle during drug transfer by a syringe (90.61%) (Figure 5A). In drug transfer by CSTD(JLY), the drug-transfer device and puncture outfit were flushed separately repeatedly to collect drugs from them. The collected drugs were added together to obtain a residual sample in the drug-transfer device of CSTD(JLY). Figure 5B reveals that the average percent residual in drug transfer by CSTD(JLY) was 0.72%, significantly lower than that in drug transfer by a syringe (1.96%). Figure 5C shows that the average percent residual of a sample in the vial upon drug transfer by CSTD(JLY) (1.96%) did not exhibit a significant difference from that of a sample in the vial upon drug transfer by a syringe. Hence, the average percent residual of a sample in a vial was equivalent between these two methods of drug transfer. Figure 5D demonstrates that the average percent leakage of a sample upon drug transfer by CSTD(JLY) (1.63%) was significantly lower than that of a sample upon drug transfer by a syringe (4.86%).

#### 3.3.3 Closed performance of CSTD(JLY) for the transfer of nimodipine

**FIGURE 6:**
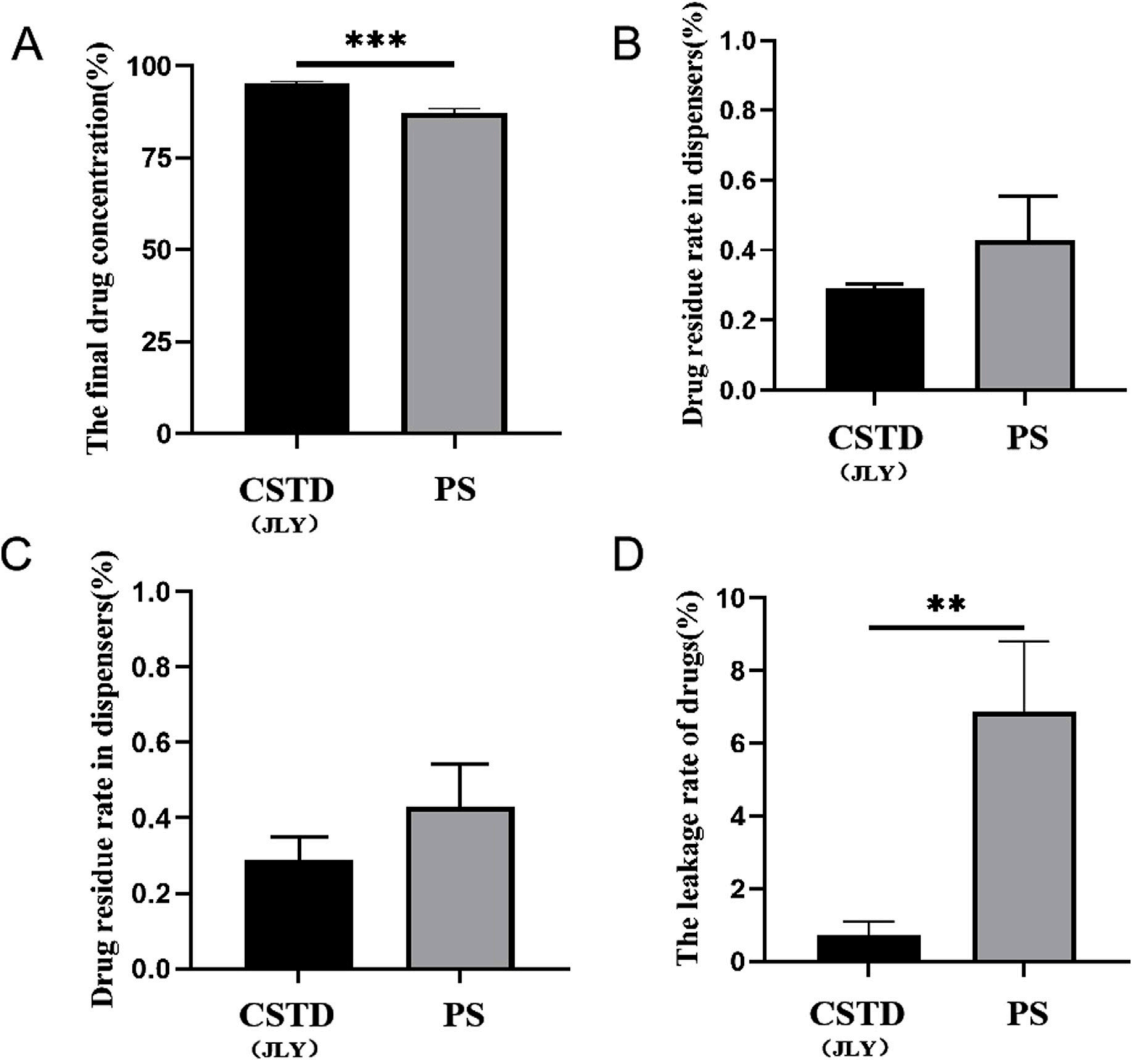
Closed performance of CSTD(JLY) and a syringe (PS) for the transfer of nimodipine: **(A)** Final concentration of nimodipine in an infusion bottle; **(B)** Percent residual of nimodipine in the drug-transfer device; **(C)** Percent residual rate of nimodipine in a vial; **(D)** Percent leakage of nimodipine; Statistically significant differences from the syringe group were indicated as **p < 0.01 and ***p < 0.001.

Nimodipine was transferred by CSTD(JLY) and a syringe separately (Figure 6). The average yield of a sample in an infusion bottle upon drug transfer by CSTD(JLY) was 95.180%, significantly higher than that of a sample in an infusion bottle upon drug transfer by a syringe (87.185%) (Figure 6A). The average percent residual in the drug-transfer device upon drug transfer by CSTD(JLY) was 0.290%, which was not significantly different from that in the drug-transfer device upon drug transfer by a syringe (0.430%) (Figure 6B). The percent residual of a sample in a vial upon drug transfer by CSTD(JLY) (3.805%) exhibited no significant difference from that of a sample in a vial upon drug transfer by a syringe (4.175%). Hence, the percent residual of a sample in a vial was equivalent between these two methods of drug transfer (Figure 6C). Figure 6D reveals that the average percent leakage of a sample upon drug transfer by CSTD(JLY) was 0.725%, significantly lower than that of a sample upon drug transfer by a syringe (6.87%), which indicated that use of CSTD(JLY) could reduce drug leakage significantly.

#### 3.3.4 Closed performance of CSTD(JLY) for the transfer of tropisetron

**FIGURE 7:**
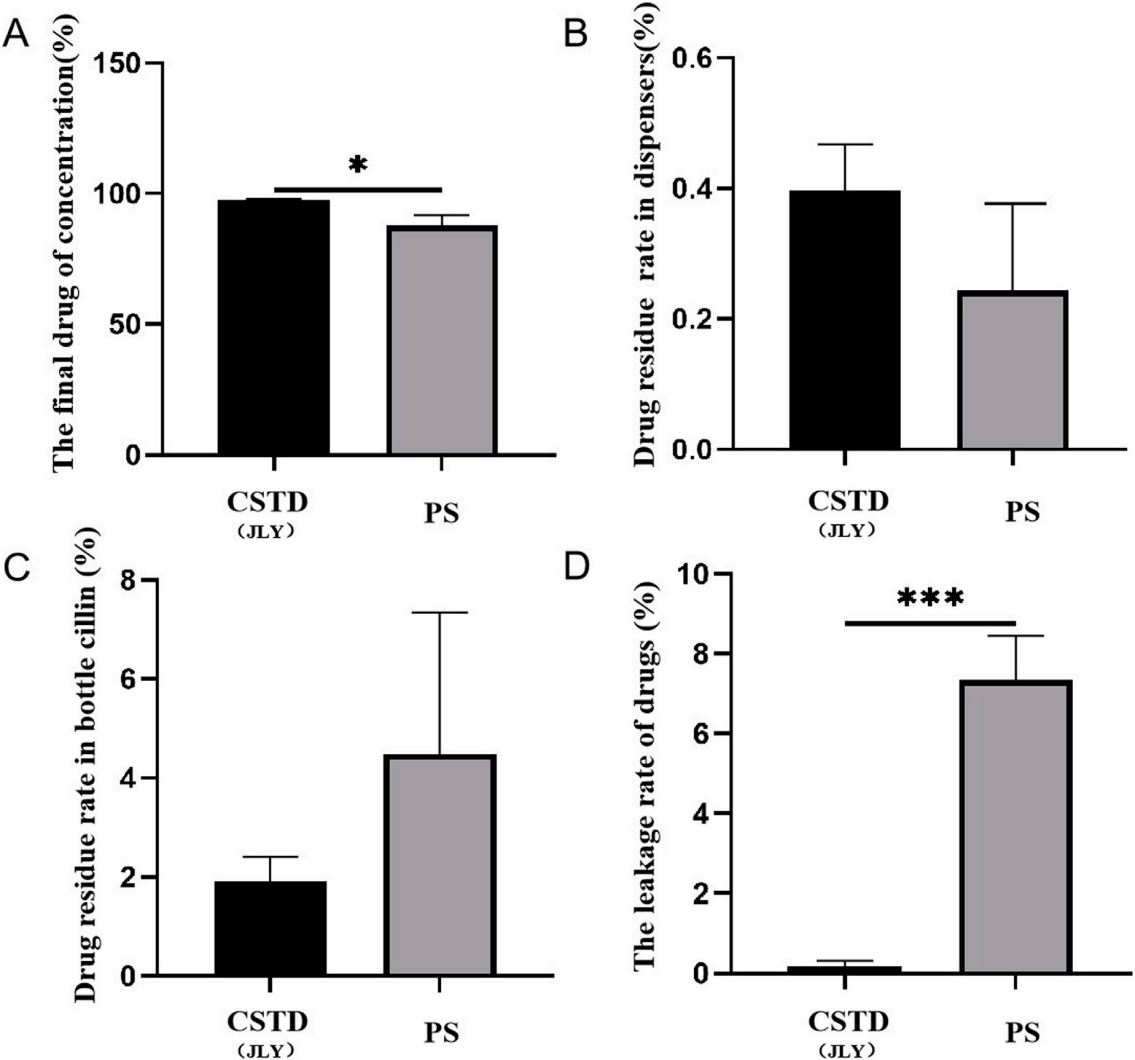
Closed performance of CSTD(JLY) and a syringe (PS) for the transfer of tropisetron: **(A)** Final concentration of tropisetron in an infusion bottle; **(B)** Percent residual of tropisetron in the drug- transfer device; **(C)** Percent residual of tropisetron in a vial; **(D)** Percent leakage of tropisetron; Statistically significant differences from the syringe group were indicated as *p < 0.05, **p < 0.01 and ***p < 0.001.

Tropisetron was transferred by CSTD(JLY) and a syringe separately (Figure 7). The average yield of a sample in an infusion bottle upon drug transfer by CSTD(JLY) was 97.52%, significantly higher than that of a sample in an infusion bottle upon drug transfer by a syringe (87.93%) (Figure 7A). The average percent residual in the drug-transfer device upon drug transfer by CSTD(JLY) was 0.39%, which was not significantly different from that in the drug-transfer device upon drug transfer by a syringe (0.91%) (Figure 7B). The percent residual of a sample in a vial upon drug transfer by CSTD(JLY) (1.903%) exhibited no significant difference from that of a sample in a vial upon drug transfer by a syringe (3.813%). Hence, the percent residual of a sample in a vial was equivalent between these two methods of drug transfer (Figure 7C). Figure 7D reveals that the average percent leakage of a sample upon drug transfer by CSTD(JLY) was 0.187%, significantly lower than that of a sample upon drug transfer by a syringe (7.347%), which indicated that drug transfer by CSTD(JLY) could reduce drug leakage significantly.

#### 3.3.5 Closed performance of CSTD(JLY) for the transfer of cyclophosphamide

Cyclophosphamide is a commonly used ACD. If healthcare workers are exposed to this hazardous agent, they may suffer asthma, birth defects, miscarriage, or cancer. The transfer of cyclophosphamide by CSTD(JLY) and a syringe were simulated in a biological safety cabinet in a PIVAS of the Affiliated Hospital of Guangdong Medical University. The residue of cyclophosphamide was analyzed by HPLC, and percent leakage of cyclophosphamide was calculated.

**FIGURE 8:**
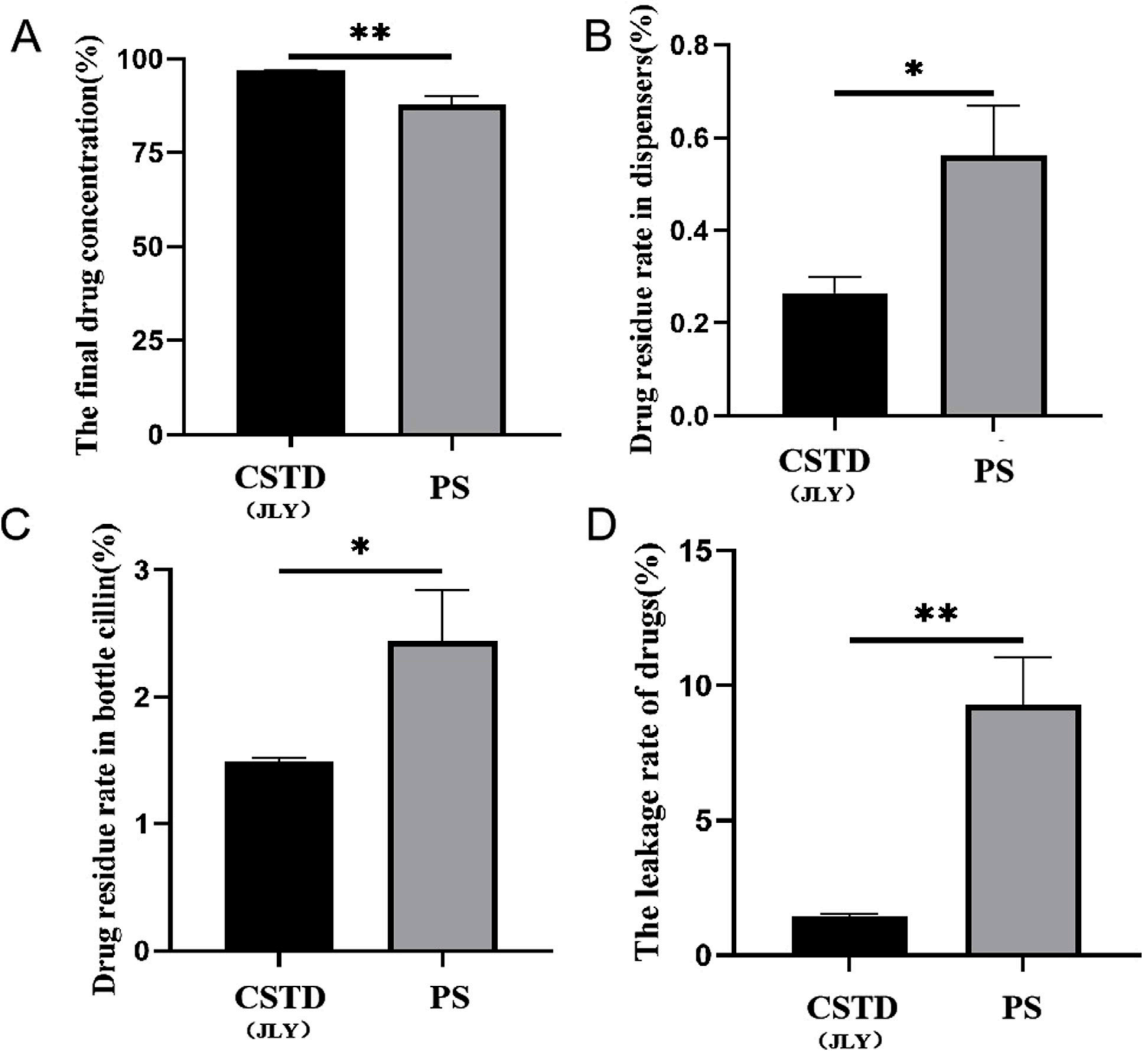
Closed performance of CSTD(JLY) and a syringe (PS) for the transfer of cyclophosphamide: **(A)** Final concentration of cyclophosphamide in an infusion bottle; **(B)** Percent residual of cyclophosphamide in the drug-transfer device; **(C)** Percent residual of cyclophosphamide in a vial; **(D)** Percent leakage of cyclophosphamide; Statistically significant differences from the syringe group were indicated as *p < 0.05, **p < 0.01 and ***p < 0.001.

Figure 8A reveals that when CSTD(JLY) was used to transfer cyclophosphamide to an infusion bottle, the yield of cyclophosphamide within the infusion bottle was 96.81%, significantly higher than that of cyclophosphamide upon drug transfer by a syringe (87.73%). The average percent residual in the drug-transfer device upon drug transfer by CSTD(JLY) was 0.27%, significantly lower than that in the drug transfer-device upon drug transfer by a syringe (0.56%) (Figure 8B). The percent residual in a vial upon drug transfer by CSTD(JLY) was 1.49%, significantly lower than that in a vial upon drug transfer by a syringe (2.44%) (Figure 8C). Figure 8D demonstrates that the average percent leakage of a sample upon drug transfer by CSTD(JLY) was 1.45%, significantly lower than that of a sample upon drug transfer by a syringe (9.27%), which indicates that drug transfer by CSTD(JLY) could reduce drug leakage significantly. It is consistent with the literature report that using the CSTD PhaSeal can significantly reduce the leakage of cyclophosphamide, compared with the standard method of drug transfer (Sessink et al., 2013; Favier et al., 2012; Clark and Sessink, 2013).

## 4 Conclusion

Compared with drug transfer by a syringe, drug transfer by CSTD(JLY) could increase the yield of drug significantly and reduce drug leakage greatly. The main reason is that CSTD(JLY) has a special structure that enables automatic pressure relief. This feature provides the most crucial function of achieving superior closed performance and relieving pressure during drug transfer.

CSTD(JLY) can decrease the resistance in the push/pull of the piston rod significantly when an operator transfers drugs, thereby reducing the burden on the hands of the operator during drug transfer. There are many interfaces in the CSTD(JLY) system to ensure that the system is highly sealed during the drug transfer process. CSTD(JLY) could prohibit the leakage of FS, common clinical drugs, and common ACDs. CSTD(JLY) could solve the problem of drug leakage during drug transfer, thereby reducing the exposure risk for healthcare workers and patients.

## Data Availability

The original contributions presented in the study are included in the article/supplementary material, further inquiries can be directed to the corresponding authors.
